# Leveraging pre-trained language models for mining microbiome-disease relationships

**DOI:** 10.1186/s12859-023-05411-z

**Published:** 2023-07-19

**Authors:** Nikitha Karkera, Sathwik Acharya, Sucheendra K. Palaniappan

**Affiliations:** 1 The Systems Biology Institute, Tokyo, Japan; 2 Iom Bioworks Pvt Ltd., Bengaluru, India; 3 PES University, Bengaluru, India; 4 SBX Corporation, Tokyo, Japan

**Keywords:** Microbe-disease relationship extraction, Language models, Fine-tuning, Deep-learning, Transfer learning, Biomedical informatics, Natural language processing

## Abstract

**Background:**

The growing recognition of the microbiome’s impact on human health and well-being has prompted extensive research into discovering the links between microbiome dysbiosis and disease (healthy) states. However, this valuable information is scattered in unstructured form within biomedical literature. The structured extraction and qualification of microbe-disease interactions are important. In parallel, recent advancements in deep-learning-based natural language processing algorithms have revolutionized language-related tasks such as ours. This study aims to leverage state-of-the-art deep-learning language models to extract microbe-disease relationships from biomedical literature.

**Results:**

In this study, we first evaluate multiple pre-trained large language models within a zero-shot or few-shot learning context. In this setting, the models performed poorly out of the box, emphasizing the need for domain-specific fine-tuning of these language models. Subsequently, we fine-tune multiple language models (specifically, GPT-3, BioGPT, BioMedLM, BERT, BioMegatron, PubMedBERT, BioClinicalBERT, and BioLinkBERT) using labeled training data and evaluate their performance. Our experimental results demonstrate the state-of-the-art performance of these fine-tuned models ( specifically GPT-3, BioMedLM, and BioLinkBERT), achieving an average F1 score, precision, and recall of over $>0.8$ compared to the previous best of  0.74.

**Conclusion:**

Overall, this study establishes that pre-trained language models excel as transfer learners when fine-tuned with domain and problem-specific data, enabling them to achieve state-of-the-art results even with limited training data for extracting microbiome-disease interactions from scientific publications.

**Supplementary Information:**

The online version contains supplementary material available at (10.1186/s12859-023-05411-z).

## Introduction

Microorganisms, in the trillions, are housed and sheltered in the human body. These microorganisms take up residence in various organs, including the gastrointestinal tract, mouth, stomach, skin, urogenital tract, and others. Their presence plays a crucial role in maintaining the host’s health and well-being [1]. Collectively, these microbes form what is known as the microbiome. Recent technological advancements have enabled us to study and quantify the microorganisms within our bodies. As a result, we can now establish both correlational and causal relationships between dysbiosis of the microbiome and disease states [2].

Structured knowledge representing the relationship between microorganisms and diseases can greatly contribute in deepening the development of microbiome-based preventive and therapeutic measures. Knowledge repositories that encompass collective information on microbiome-disease associations is usually constructed based on evidence from scientific publications. These manually curated knowledge bases are frequently utilized for downstream analysis and discovery. Several endeavors, such as Amadis [3], Disbiome [4], MicroPhenoDB [5], The Virtual Metabolic Human database [6], MADET [7], gutMDisorder [8, 9], HMDAD [10], mBodyMap [11], have focused on at cataloging and organizing this information. While these knowledge bases are of high quality, their construction is a labor-intensive and expensive process due to the substantial manual effort required for curation. Furthermore, keeping these databases up-to-date poses significant challenges, particularly given the rapid pace of microbiome research and the continuous accumulation of new findings. To provide perspective, a search for the keyword “microbiome” on PubMed returns over 140, 000 abstracts, with more than 27, 000 abstracts published in 2022 alone.

Natural Language Processing (NLP) techniques have emerged as a promising approach to effectively handle the vast amount of scientific literature. These methods enable automated analysis of extensive scientific texts and the extraction of relevant information, which can then be stored in knowledge bases. In recent years, the field of NLP has witnessed substantial advancements owing to the emergence of Large Language Models (LLMs) and Generative AI models [12]. Consequently, there has been a growing interest in leveraging these techniques to tackle problems in the microbiome field as well [13, 14]. Of particular significance is the work presented by Badal et al. [13], which highlights the key challenges that must be addressed to establish meaningful knowledge bases for the microbiome disease problem. This research provides valuable insights into the nuances and intricacies of the problems in this subdomain and serves as a foundation for our work too.

To address the specific challenge of extracting associations between diseases and the microbiome using NLP techniques, the solution skeleton is usually a combination of the following steps:

- Identifying disease and microbe mentioned in scientific texts. This step typically involves utilizing algorithms such as Named Entity Recognizers (NERs), linguistic taggers, and dictionaries to locate disease and microbe references within each document or sentence.
- Establishing the existence of a relationship or association between pairs of diseases and microbes. Relationship extraction algorithms are commonly employed for this task. Extensive research has been conducted in this area, with notable contributions so far [10, 15–19].
- Once the presence of a relationship is established, determining the nature of the relationship. For example, investigating whether the presence of a specific bacterium is positively correlated with a particular disease. Specialized relationship extraction algorithms are employed to address this, which is also the primary focus of this paper.

### Related work

At its core, the current problem is that of relation extraction which has decades of prior work in the Bio-NLP domain [20, 21]. However, the traditional models for relation extraction are now being surpassed by deep learning based NLP models [22] which are shown to be superior in their performance.

In terms of related work to the disease-microbiome extraction task, two main works are related to ours. First, Park et al. [23], proposed an ensemble model for this problem. Their approach involves two steps: first, a relation detection model based on Hierarchical Long Short-Term Memory (LSTM) networks to determine the presence of a disease-microbe relationship. Second, they extract the specific relation type by employing a substantial collection of rule sets or patterns, amounting to around 1000. However, this approach has limitations as it requires manual maintenance of the rule list for relation extraction, making it impractical for large-scale efforts.

Next is the work by Wu et al[24] that focuses on a deep-learning strategy for solving this problem. Their approach first involves preparing training datasets. For this, they start by collecting a large corpus of text from PubMed related to microbiome and diseases and subsequently employ Named Entity Recognition (NER) tools to identify microbe and disease entities within the text. Next, they manually create two corpora for microbe-disease interactions: a high-quality gold-standard corpus (GSC) and a silver-standard corpus (SSC) that is known to contain errors. These corpora are then used as training data. Subsequently, they utilize a deep-learning-based relation extraction algorithm [25] to train a deep-learning model using the GSC data which did not yield the best results. Subsequently, they implement a 2-step learning process, where the model is first trained on the error-prone SSC corpus and then fine-tuned using transfer learning on the GSC corpus. The authors report that this 2-step approach significantly improves the accuracy of relationship extraction, achieving an F-score of 0.7381. For our problem statement, this result represents the current state-of-the-art as reported in the scientific literature. While the approach holds interest, we theorized that the expensive training of deep-learning models could be avoided by directly fine-tuning pre-trained models, in addition to improved accuracy gains.

### Our contribution

Our paper offers multiple contributions. Firstly, we recognize the significant advancements in the field of large language models such as GPT-3 [26], BERT [27] etc. Leveraging the power of these models, we utilize them in our task to achieve state-of-the-art results. The utilization of deep learning and transformer models allows us to effectively capture complex patterns and relationships within the literature. Secondly, our approach highlights the relevance of deep learning and transformer models in reducing the requirement for large amounts of training data. In contrast to the study by [24] or other deep learning models, our method benefits from transfer learning and task-specific fine-tuning. This advantage enables us to achieve excellent results with a smaller amount of training data, making our approach efficient and practical. Furthermore, our approach can handle cases where disease-microbe relationships span multiple sentences. Deep learning and transformer models have the capacity to capture long-range dependencies and contextual information, allowing us to effectively address the complexity of such relationships.

To tackle the task of relationship extraction, we adopt two solution strategies. First, we treat it as a classification (discriminative) task. Here, the model receives the evidence describing the relationship between a disease and a microbe as input, along with a question probing their relationship. The model’s output is expected to provide the answer from one of the four labels: positive, negative, relate, or NA. This approach enables us to utilize the discriminative power of deep learning and transformer models.

Next, we reformulate the task as a generative text task. In this setup, we provide the model with evidence describing the relationship between a disease and a microbe, as well as a question as a prompt. The model is then tasked with generating the correct label. This generative approach leverages the expressive nature of deep learning and transformer models to generate informative and accurate labels.

For further details on our methodology and experimental setups, we provide comprehensive information in the subsequent sections of our paper, highlighting the specific ways in which deep learning and transformer models contribute to the success of our approach.

**Fig. 1:**
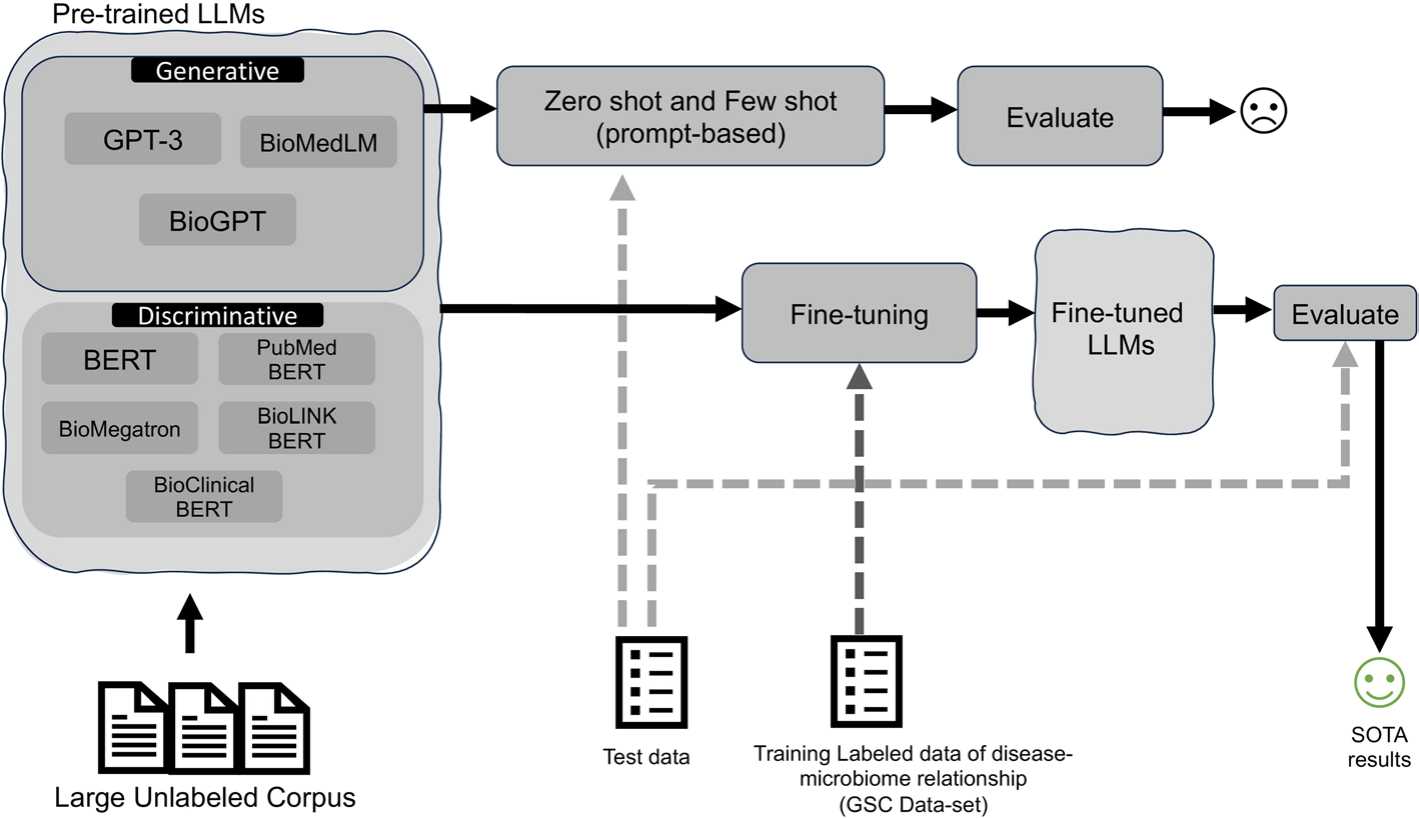
Our contributions and study design for extracting disease-microbiome relationships

To establish baselines, we employ various state-of-the-art models, both domain-independent and domain-specific. Initially, we explore the potential of pre-trained language models as zero-shot and few-shot learners, without fine-tuning, potentially eliminating the need for training data or specialized retraining and fine-tuning. Subsequently, we fine-tune these models using curated training data, obtained from [24], to further enhance their capabilities.

Firstly, among the generative models, BioGPT [28], BioMedLM [29] and GPT-3 [26] were considered. These models are designed to generate/complete the response based on the given question and context(prompt). They were a natural choice for our task, as they are known to perform well in zero-shot or few-shot learning scenarios. Additionally, we incorporated the following language models in the discriminative setting: BERT [30], ClinicalBERT [31], PubMedBERT [32], BioMegatron [33] and BioLinkBERT [34]. With these models, the objective was for the model to classify the input into one of the four predefined labels. Details of our study and the summary of the outcomes are shown in Fig. 1.

Our experiments show that these models fall short in the zero-shot or few-shot setup, highlighting the need for domain-specific fine-tuning and task-specific training data. After fine-tuning, among the generative models, GPT-3 performed well. However, we observed that these models sometimes produced varying outputs for the same prompt, owing to their generative nature, which can pose challenges. Among the discriminative models, the model fine-tuned on BioLinkBERT consistently yielded the best results among the tested models. The next section formally introduces the problem and the solution strategy.

## Problem formulation and models considered

Our objective is to extract and determine the relationships between disease and microbe terms from natural language text. Formally, given a scientific text *T* and a pair of entities (*e*1, *e*2) occurring in *T*, where $e1 \in D$ and $e2 \in M$, with *D* and *M* representing the sets of all disease and microbe names, respectively, the task is to predict a label *y* that represents the relationship between *e*1 and *e*2. The label *y* belongs to the set $\{positive, negative, relate, NA\}$.

One approach to mathematically formulate this problem is by using a supervised learning method. In this approach, the model is trained on a dataset consisting of labeled sentences and entities. The model learns a function *f* that maps the scientific text and entity pair (*T*, *e*1, *e*2) to a predicted label *y*, such that $y = f(T, e1, e2)$. The function *f* can be realized using various machine-learning techniques and models.

In the context of microbe-disease relationships, the following labels are defined [24]:

- (positive): This label indicates a positive correlation between the microbe and the disease. It implies that the microbe can worsen the disease or that its presence increases when the disease occurs.
- (negative): This label indicates a negative correlation between the microbe and the disease. It suggests that the microbe can act as a treatment for the disease or that its presence decreases when the disease occurs.
- (relate): This label indicates a relationship between the microbe and the disease without additional information about whether it was positive or negative. It signifies that they appear to be associated with each other. In a sense, this label can be considered a super-set of positive and negative labels.
- (NA): This label indicates that the microbe and the disease mentioned in the text are not related to each other.

**Fig. 2:**
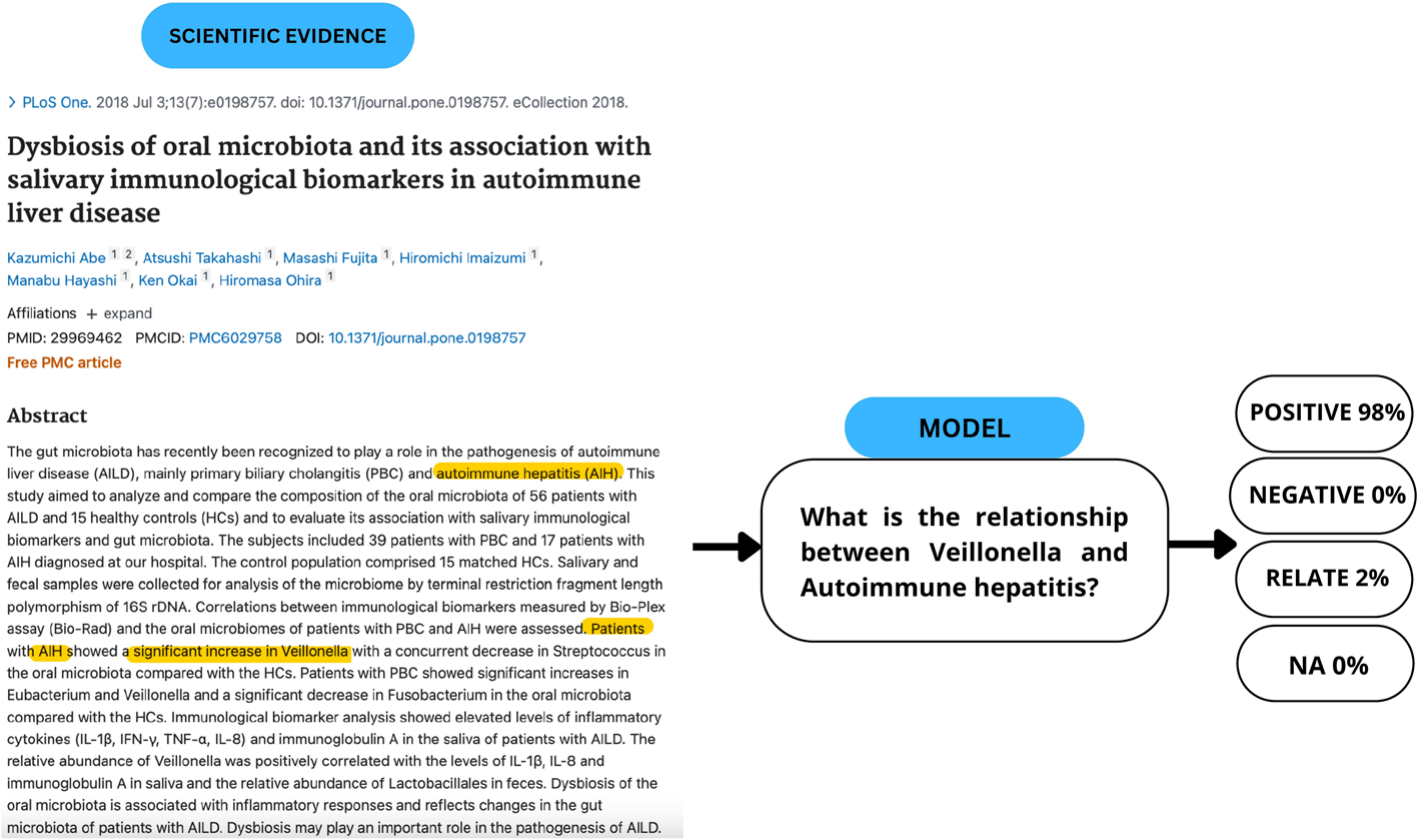
Problem formulation for inferring microbe-disease relationship

In our case, given the same scientific text *T* and entities of interest (*e*1, *e*2), and a question posed as follows: “What is the relationship between *e*1 and *e*2?” our models are expected to provide an answer from the set *positive*, *negative*, *relate*, *NA*. The problem formulation in our setting is illustrated in Fig. 2.

### Pre-trained language models considered

Now, we describe the various pre-trained models that were leveraged for fine-tuning in this study. The choice of models was based on best-in-class for biomedical domain specific tasks.

#### Generative setting

*BioMedLM 2.7B* [29] is a large language model trained on a dataset of biomedical literature and is based on GPT-2 model. It has 2.7 billion parameters. It is effective for a variety of tasks, including natural language inference, question answering, and text summarization. BioMedLM 2.7B is a valuable tool for researchers and clinicians who need to access and process biomedical information.

*BioGPT* [28] is a domain-specific generative pre-trained transformer language model for biomedical text generation and mining. It is trained on 15 million PubMed abstracts and has 1.5 billion parameters. It was developed by Microsoft Research and is based on the GPT-2 language model.

*GPT-3* [26], or Generative Pre-trained Transformer 3, is a state-of-the-art language model developed by OpenAI. With 175 billion parameters, it exhibits remarkable proficiency in generating coherent and contextually relevant text across various domains. The most competent model available in OpenAI is “text-davinci-003,” while there are other models as well. The prompt used for this experiment is detailed in the supplementary website [see Additional file 1]. For all experiments, we changed the Temperature parameter to 0 making the outputs less random.

#### Discriminative setting

*BERT*(Bidirectional Encoder Representations from Transformers) model [27] is among the most well-known and early LLMs based on transformer architectures. It was specifically trained on Wikipedia and Google’s BooksCorpus. BERT is known to be a very good general-purpose model that works well for most language tasks. In our case, we used BERT first to see if generic models could perform well for our task before resorting to domain-specific adaptations. For our experiments, we used the “Bert-base-uncased” model from the Hugging Face library [35].

*PubMedBERT* is a BERT-based model pre-trained from scratch using 14 million abstracts from PubMed. It consistently outperforms all the other BERT models in most biomedical NLP tasks, often by a significant margin as reported in [32]. Specifically, microsoft/BiomedNLP-PubMedBERT-base-uncased-abstract-fulltext is the pre-trained model that was utilized.

*BioMegatron* models are pre-trained from scratch on the PubMed dataset. A large biomedical language model pre-trained on a large literature corpus is an excellent starting point for identifying the microbiome-disease relation type. The pre-training of this model takes PubMed abstracts and full-text commercial-collection (CC) that are free of copyrights. When compared to the prior state-of-the-art (SOTA), BioMegatron significantly outperformed across a variety of tasks. In contrast to models pre-trained on wide domain datasets, [33] demonstrates that language models specialized for a particular domain perform at their best.

*BioLINK-BERT* model is trained on abstract data, similar to PubMedBERT, but with the addition of citation links between articles [34]. Unlike previous works, which only use their raw text for pre-training, academic papers have extensive dependencies on one another through citations (references). Incorporating citation links assists language models in learning the dependencies between papers and the knowledge that spans them.

*BioClinicalBERT* [36] is a BERT-based language model that has been pre-trained on a dataset of 880 million words of biomedical and clinical text which allows it to better understand and generate text from both domains. It is a further development of BioBERT. BioClinicalBERT is available through the Hugging Face Transformers library. It is designed to improve the performance of biomedical natural language processing (NLP) tasks, such as named entity recognition, and relation extraction.

**Fig. 3:**
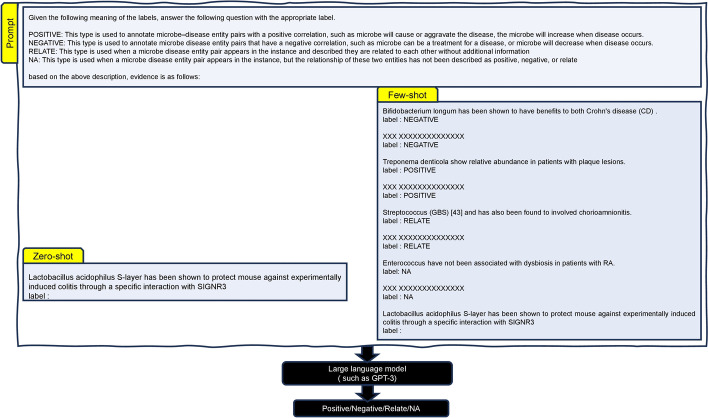
Zero-shot and few-shot learning setup for inferring microbe-disease relationship using GPT-3. The Ground truth for this example is “negative”. However GPT-3 returns “relate” in zero-shot and “positive” in few-shot setting

## Poor performance in zero-shot and few-shot setting

We first investigated the performance of generative language models in a zero-shot and few-shot learning setting. We evaluated all three models, the details of the prompts used are detailed in the supplementary website [see Additional file 1].

Zero-shot learning allows the model to extract relationships between disease and microbiome without requiring specific training dataset for those relationships. By leveraging the pre-trained knowledge and semantic understanding encoded within the language model, the model can generalize and infer relationships based on the provided input. This approach is particularly valuable when dealing with unseen relationship types, as it enables the model to make predictions even for relationships it has not encountered during training.

On the other hand, few-shot learning extends the capabilities of zero-shot learning by enabling the model to learn from a limited number of labeled examples for a specific relationship. Rather than relying solely on pre-encoded knowledge, few-shot learning allows the model to adapt and make accurate predictions using the additional labeled data. By leveraging both the pre-trained knowledge and the limited labeled examples, few-shot learning enhances the model’s ability to generalize and extract relationships, even in scenarios where labeled data is scarce or new relationship types are introduced. We use a “two-shot-learning” setup to infer the microbe-disease relationship. More specifically, two examples of scientific text per class along with its annotation were provided for each of the labels as shown in Fig. 3. A natural language description is also provided along with the prompt for the model to learn about the task as this often improves the model’s performance (See [26, 37].

### Experimental results

In summary, for the zero-shot setting, we found that the performance of the models was poor as shown in Table 1. For GPT-3, the outputs are generated within the four labels, but they don’t follow the same casing for every predicted label. It achieved a f1-score of 0.5 with low precision. A detailed analysis of the results showed that the model performs poorly for the “NA” class. Details can be found in the supplementary website [see Additional file 1]. Surprisingly, BioMedLM and BioGPT did not even produce sensible outputs.

Even in the few-shot setting, we noticed that there were only marginal improvements in the results with GPT-3 (f1-score of 0.56), while no tangible outputs were generated for BioMedLM and BioGPT as shown in Table 1. It was clear that despite the general observation that generative models provide good outcomes in zero or few-shot learning settings, performance still depends on the task-specific domain. Similar outcomes have been established previously [38]. For our problem, using models out of the box was of limited utility, this could also be due to the counterintuitive definition of positive and negative labels. For all experiments in this section, default model parameters were used.

**Table 1:** Performance metrics of the GPT-3 model in the zero-shot and few-shot setting

Model	Accuracy	F1 score	Precision	Recall
GPT-3 (zero-shot)	0.48	0.50	0.58	0.48
GPT-3 (few-shot)	0.57	0.56	0.57	0.57

## Dataset for fine-tuning: considerations for improved accuracy

**Fig. 4:**
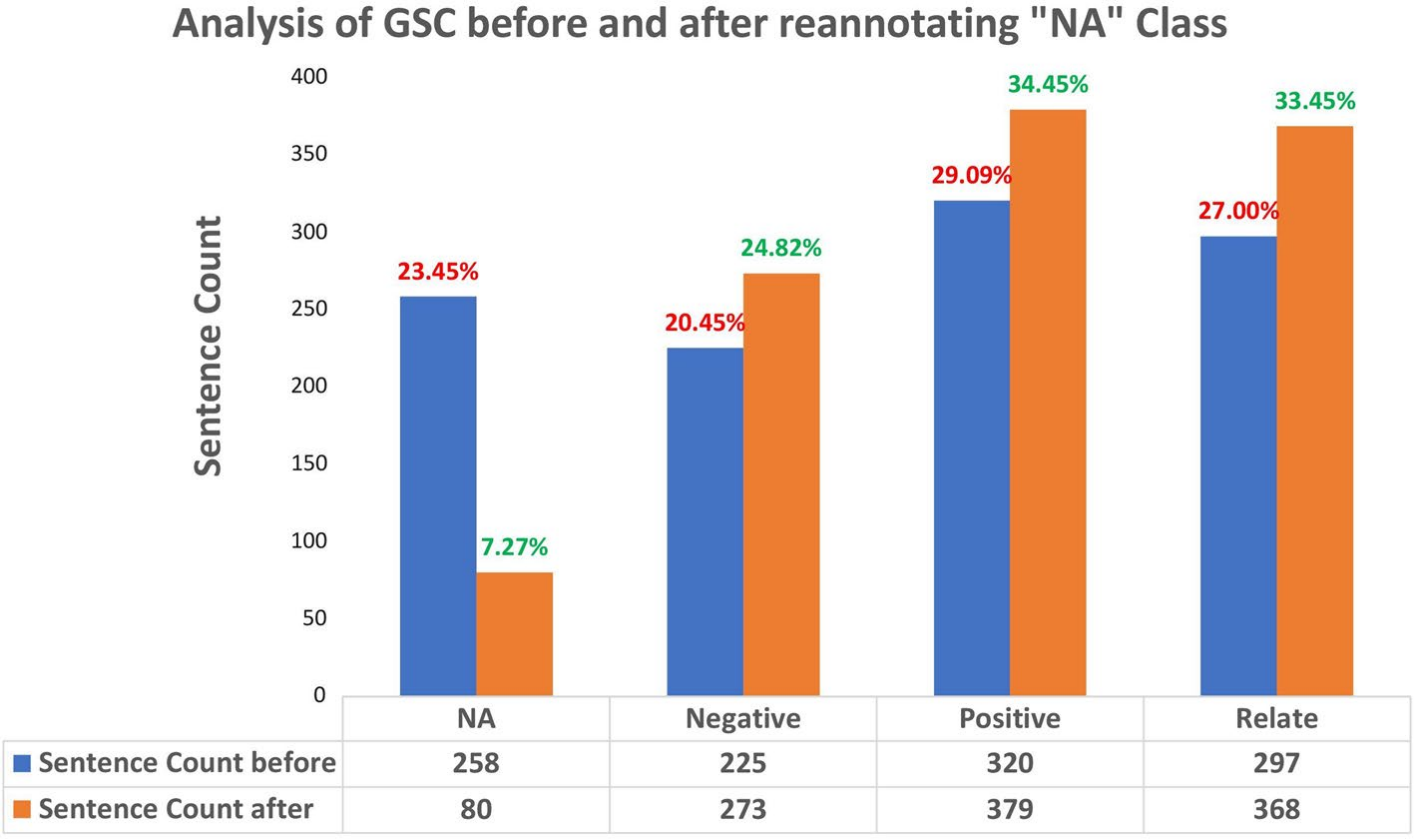
Distribution of relation types for GSC after correcting for issues in “NA” relation

To fine-tune the various language models for our task, we utilized the human-annotated gold-standard corpus (GSC) from [24]. This dataset consists of 1100 sentence instances that describe interactions between the microbiome and diseases, along with corresponding labels of “positive,” “negative,” “relate,” and “NA.” These sentences were selected by employing a semi-automated pipeline to identify diseases and microbes mentioned in articles from PubMed and PubMed Central using the keyword “microbe”. Expert annotators then reviewed each sentence and assigned them to one of the four categories. For a comprehensive understanding of how the GSC dataset was constructed, we refer interested readers to [24]. Out of the 1100 sentences, the distribution of classes is depicted in shown in Fig. 4. This dataset served as the basis for fine-tuning our pipeline.

Interestingly, we identified a significant number of labeling errors in the GSC dataset, particularly for the “NA” category. To address this issue, we re-annotated the statements with two postdoctoral-level researchers who re-labeled all the sentences initially marked as “NA”. The annotation process was facilitated using the Doccanno tool [39]. Each researcher independently re-annotated all the “NA” labels, followed by a collaborative review of each other’s annotations. Through consensus, a final label was agreed upon for each sentence.

Our analysis revealed that out of the original 258 sentences labeled as “NA” in the GSC dataset, 178 sentences were found to be mislabeled. In the supplementary website [see Additional file 1], we provide illustrative examples and a comprehensive list of the re-annotated data. As depicted in Fig. 4, there was a substantial decrease in the number of training data points for the “NA” category, accompanied by an increase in samples for the negative, positive, and relate categories. All further fine-tuning was performed on the corrected dataset. This dataset is available for review in the supplementary website [see Additional file 1].

## Domain specific fine-tuning of different language models

We fine-tune all the models using the dataset outlined in the preceding section for the task at hand. Notably, the methodology employed by [24] involved a two-step approach. In the first step, an error-prone silver training corpus was utilized to train a relation extraction algorithm [25]. Subsequently, the model trained in the initial step was employed in conjunction with transfer learning techniques for fine-tuning with the GSC dataset. The authors demonstrated that this two-step transfer learning process yielded state-of-the-art (SOTA) results. Although this approach is interesting, we hypothesized the costly training of deep-learning models could be circumvented by directly fine-tuning pre-trained models. In essence, our strategy is to take models that were previously trained on extensive generic or domain-specific free text as the foundation, and subsequently fine-tune them specifically for the targeted problem using a minimal quantity of high-quality training data (in our case, GSC).

### Methodology

**Fig. 5:**
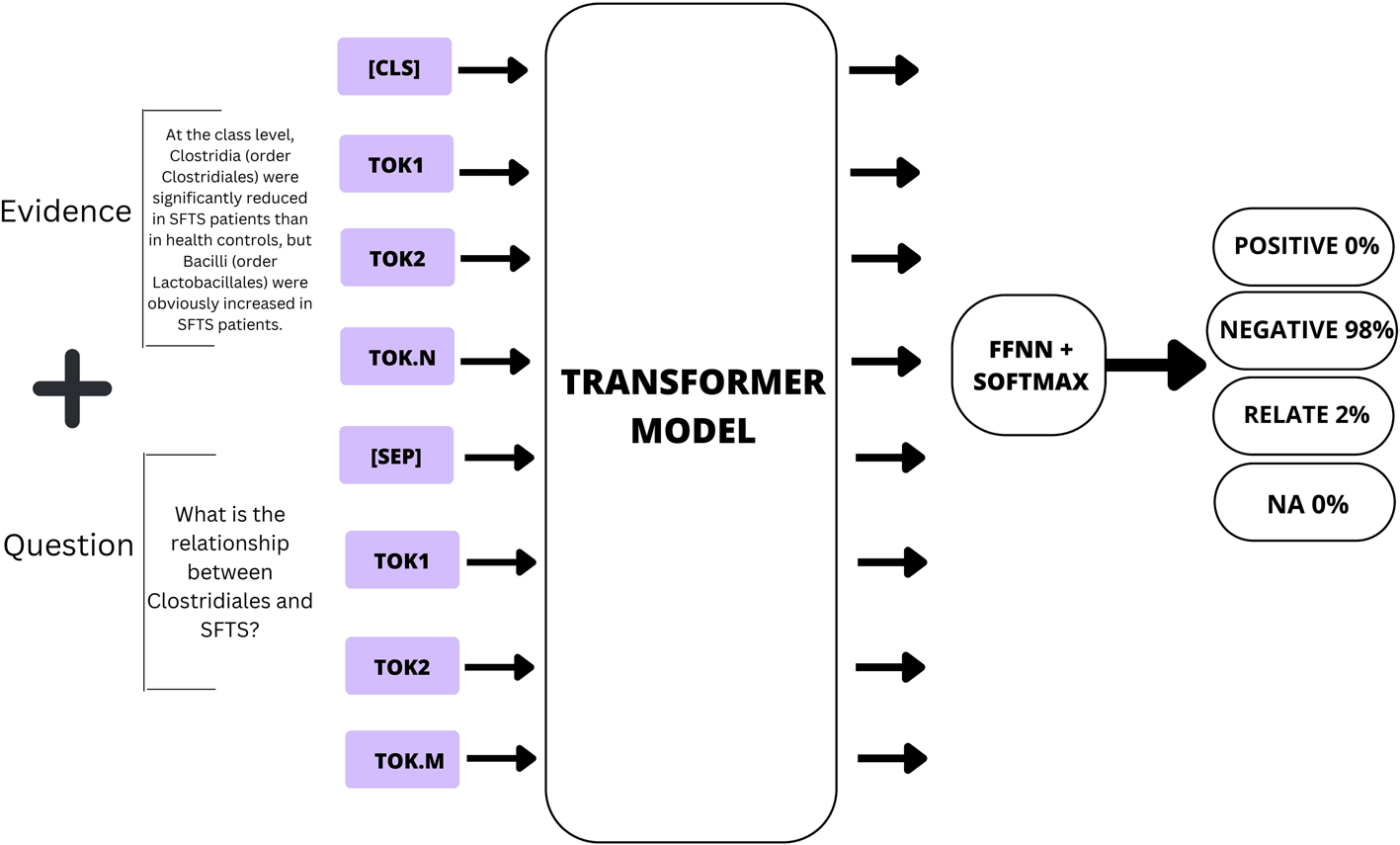
Illustration and mapping of the evidence and question tokens into the models for our discriminative class of models

Figure 5 shows the mechanism in which the evidence-question are taken in pairs for the fine-tuning purpose for the discriminative class of models namely BERT [30], BioMegatron [33], PubMedBERT [32], BioClinicalBERT [28] and BioLinkBERT [34]. For this, we resort to a typical tokenizing procedure of the evidence-question pair to produce token IDs and the attention mask. A maximum sequence length of 512 is maintained, as this is what BERT-based models are limited to. For all base models considered, during the fine-tuning process, a learning rate of $5e-5$, a weight decay of 0.01, number of epochs=7, and Adam’s optimizer with a layer-wise learning rate decay of 0.9 was applied. All models were trained using an NVIDIA GeForce RTX 2080 Ti with 12GB memory, 16 CPUs, and 64GB memory. The fine-tuning process took about 30 min for each model.

Among the generative models, GPT-3 model was fine-tuned using the OpenAI API of the GPT-3 *davinci* model. Details of the fine-tuning process are available on the OpenAI website (link: https://openai.com/api/). The inference parameters for the fine-tuned model are t=0.0, top_p=1, max_tokens=1 with other parameters with its default settings. BioMedLM’s stanford-crfm/BioMedLM model was fine-tuned on an A40 GPU instance with deepspeed setting for efficiency. This helped in training the model with  18GB GPU memory utilization. The model was trained for 20 epochs with batch_size=2, gradient_accumulation_steps=2, learning_rate=2e-06 with other parameters kept the same as their default settings. It took around 7hrs to complete 20 epochs. Similarly, BioGPT was fine-tuned on a single NVIDIA GeForce RTX 2080 Ti with 12GB memory. The model was trained using parameters similar to DDI (Drug-Drug Interaction) experiment in BioGPT [28] for Relation extraction purposes.

### Data preparation for fine-tuning

This section provides details of the data processing and prompt design for the fine-tuning process of different models.

#### Discriminative models

The training data format for fine-tuning these models follows a simple structure. Each example consists of an input and an output. The input is represented by two strings, an Evidence String and a Question String, separated by a delimiter. For example, the Evidence string can be “Additionally, some members of the phylum such as Faecalibacterium prausnitzii, a member of the Clostridiales-Ruminococcaceae lineage have been shown to have anti-inflammatory effects due to the production of the short-chain fatty acid butyrate and have been negatively correlated with inflammatory bowel disease.” and Question as “What is the relationship between inflammatory bowel disease and Clostridiales ?”. The output corresponds to the target label associated with the given input as shown below.
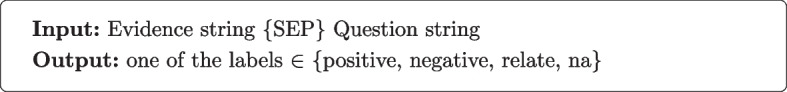


This training data format allows for a straightforward mapping between the input evidence and question, and the corresponding output label. By fine-tuning the pre-trained models on the GSC dataset encoded as above, the model can learn to effectively understand the relationship between the evidence and question, and generate accurate labels or predictions based on the input provided.

#### Generative models

The training data format for GPT-3 model consists of a collection of examples, each represented by a prompt and a completion string that corresponds to the label.

The “prompt” key corresponds to the text that serves as the input or context for the model. It contains evidence related to the microbiome and disease relationship. In this format, the prompt text is structured as the evidence string followed by a question string, separated by a line break (“\n”). The evidence string provides the background or supporting information, while the question string represents the specific question to be answered by the model. For example, a prompt can be:

* “Evidence: Additionally, some members of the phylum such as Faecalibacterium prausnitzii, a member of the Clostridiales-Ruminococcaceae lineage have been shown to have anti-inflammatory effects due to production of the short-chain fatty acid butyrate and have been negatively correlated with inflammatory bowel disease107.\n Question: What is the relationship between inflammatory bowel disease107 and Clostridiales ?\n\n####\n\n”. * Here, the ending string “\n\n####\n\n” acts as a fixed separator to the model. For inference, the prompts are designed in the same format as the training dataset including the same separator with the same stop sequence to properly truncate the completion. 
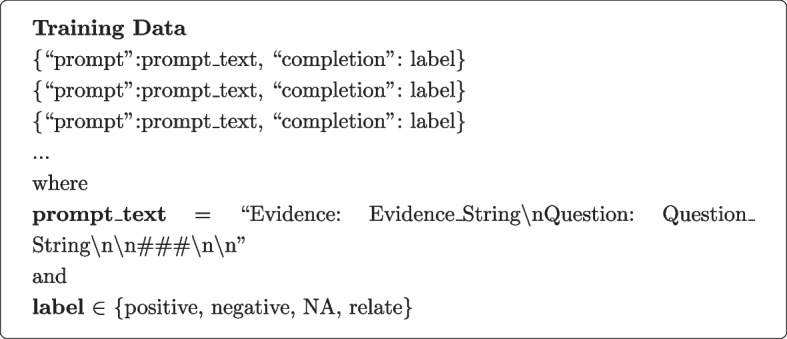


The training data format allows for multiple examples to be included, each following the same key-value structure. The detailed prompts for BioMedLM and BioGPT which are very similar to the GPT-3 prompts can be found on the supplementary website [see Additional file 1].

**Table 2:** Performance metrics different fine-tuned language models

	Model	Accuracy	F1 score	Precision	Recall
Baseline	BERE_TL(MDI)	NA	0.738	0.736	0.740
Our models (fine-tuned)	Bert-base-uncased	$0.733 \pm 0.018$	$0.731 \pm 0.015$	$0.742 \pm 0.02$	$0.733 \pm 0.018$
	BioMegatron	$0.778 \pm 0.008$	$0.769 \pm 0.013$	$0.771 \pm 0.013$	$0.778 \pm 0.008$
	PubMedBERT	$0.782 \pm 0.022$	$0.778 \pm 0.019$	$0.783 \pm 0.021$	$0.782 \pm 0.022$
	BioClinicalBERT	$0.729 \pm 0.032$	$0.724 \pm 0.029$	$0.731 \pm 0.032$	$0.729 \pm 0.032$
	**BioLinkBERT-base**	${\textbf {0.811}} \pm {\textbf {0.029}}$	${\textbf {0.804}} \pm {\textbf {0.036}}$	${\textbf {0.813}} \pm {\textbf {0.034}}$	${\textbf {0.811}} \pm {\textbf {0.028}}$
	BioMedLM	$0.806 \pm 0.028$	$0.804 \pm 0.028$	${\textbf {0.822}} \pm {\textbf {0.030}}$	$0.806 \pm 0.028$
	BioGPT	$0.732 \pm 0.017$	$0.726 \pm 0.017$	$0.732 \pm 0.025$	$0.736 \pm 0.016$
	**GPT-3**	${\textbf {0.814}} \pm {\textbf {0.021}}$	${\textbf {0.810}} \pm {\textbf {0.025}}$	$0.810 \pm 0.021$	${\textbf {0.814}} \pm {\textbf {0.021}}$

Bold indicates the performance of the models which gave the best performance

### Results

To mitigate the risk of overfitting, model performance was evaluated using a 5-fold cross-validation strategy. The curated dataset was divided into five equal parts, referred to as folds. In each iteration, the models were fine-tuned using four folds for training and evaluated on the remaining fold. This process was repeated five times, with each fold serving as the test set once. The average of the five test scores was calculated to provide the final metrics of the model. We assessed the performance using several metrics, including Accuracy, Weighted Average F1 score, Precision, and Recall, which align with those reported in [24]. The detailed results are presented in Table 2. The results of the study by [24] are shown in the table using the notation $BERE_{TL} (MDI)$. The reported f1-score reached a peak value of 0.738 along with closely aligned precision and recall scores. These results serve as our baseline.

**Fig. 6:**
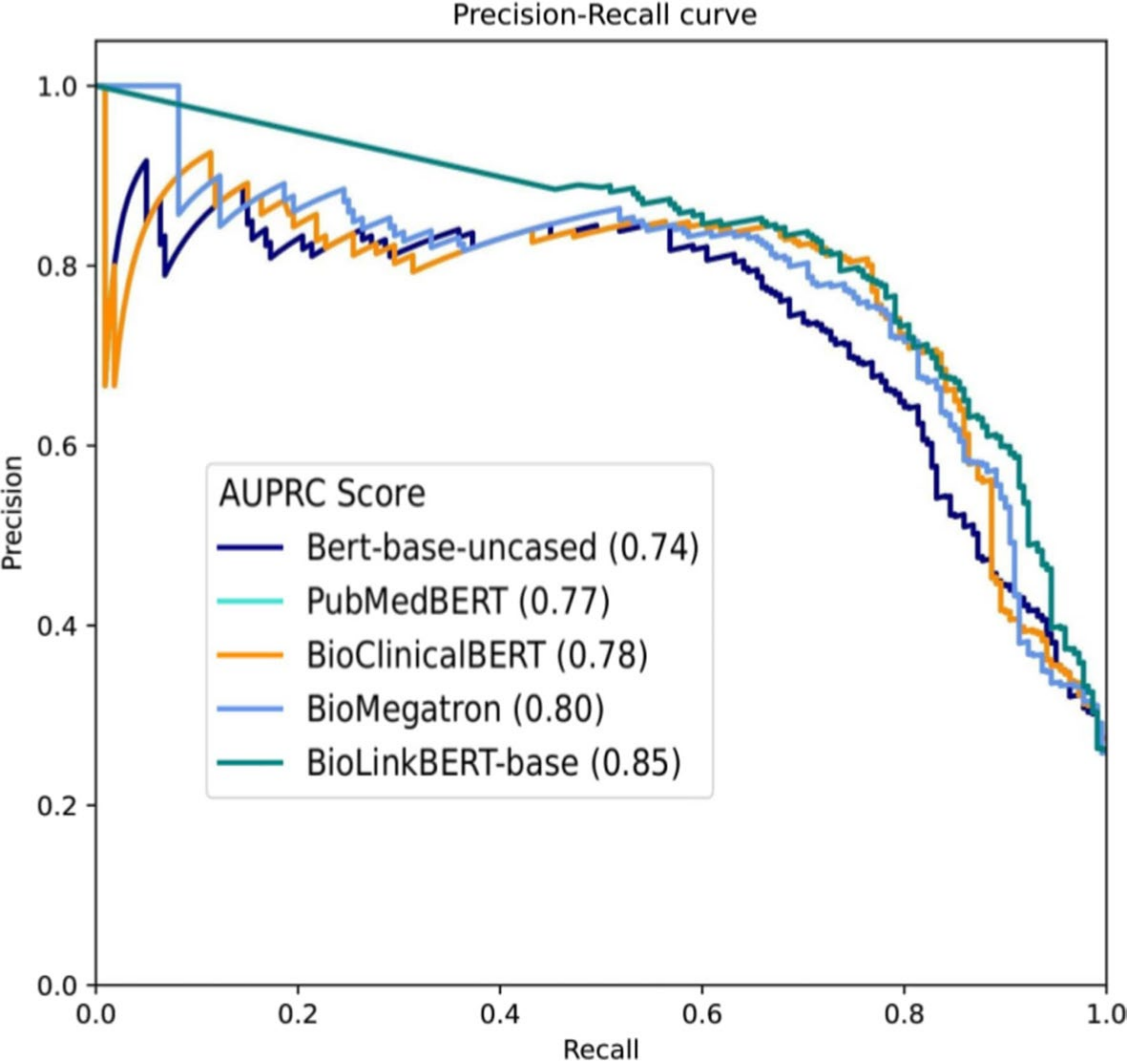
Precision recall curve for the various fine-tuned pre-trained models used in discriminative setting

Coming to the performance of the discriminative fine-tuned models, we observed significant improvements across the entire spectrum. Notably, the model trained on BioLinkBERT-base yielded the best results, achieving an average F1-score of $0.804 \pm 0.0362$ in a 5-fold cross-validation setup. Detailed information regarding all the models and the fine-tuning parameters can be found on our supplementary website (see Additional file 1). Further, to understand the characteristics of the classifier better, we plotted the precision-recall curves as shown in Fig. 6. Notably, the area under the curve for BioLinkBERT-finetuned outperformed others, reaching 0.85, indicating the best performance.

Among the generative class of models, we found that the fine-tuned GPT-3 model yielded the best overall results, as shown in Table 2. However, in terms of precision, the model fine-tuned on BioMedLM performed well as shown in Table 2. However, we noticed a few observations regarding the use of these generative models. Firstly, we sometimes noticed variability in the results with each run of the model depending on the parameters used. There were also instances where the model produced empty outputs. Additionally, since these models are generative in nature, the outputs and probabilities generated by the model do not always align with well-defined class labels. This aspect further hinders our comprehension of how these models operate and raises concerns about the reliability of their outputs. Due to these limitations, we were unable to generate a precision-recall curve for GPT-3.

**Table 3:** Per class metrics for BioLinkBERT fine-tuned model

Class	Precision	Recall	F1-score
NA	$0.556 \pm 0.131$	$0.447 \pm 0.186$	$0.457 \pm 0.096$
Negative	$0.827\pm 0.022$	$0.894\pm 0.043$	$0.859\pm 0.025$
Positive	$0.831\pm 0.015$	$0.854\pm 0.048$	$0.841\pm 0.027$
Relate	$0.829\pm 0.040$	$0.798\pm 0.053$	$0.811\pm 0.025$

**Table 4:** Per class metrics for GPT-3 fine-tuned model

Class	Precision	Recall	F1-score
NA	$0.570 \pm 0.112$	$0.366 \pm 0.108$	$0.431 \pm 0.089$
Negative	$0.847 \pm 0.047$	$0.881 \pm 0.042$	$0.863 \pm 0.041$
Positive	$0.819 \pm 0.045$	$0.866 \pm 0.034$	$0.841 \pm 0.022$
Relate	$0.820 \pm 0.027$	$0.815 \pm 0.022$	$0.817 \pm 0.015$

To gain deeper insights into the performance of the classifier and generative model, we analyzed the per-class performance metrics for both the fine-tuned generative models (GPT-3) and the discriminative models (BioLinkBERT model). As expected, the metrics for the negative, positive, and relate classes exhibited satisfactory results. However, we observed poor performance in the NA class for both the fine-tuned GPT-3 (refer to Table 4) model and the BioLinkBERT model (refer to Table 3). This deficiency in performance also accounts for the lower overall classification performance. There are two possible reasons for this outcome. Firstly, as previously discussed, the distribution of data in the dataset is imbalanced, with a smaller number of NA samples. Secondly, there may be inherent challenges in defining the classes in the original problem, which could necessitate further investigation and deliberation. However, exploring these concerns is beyond the scope of this paper.

**Fig. 7:**
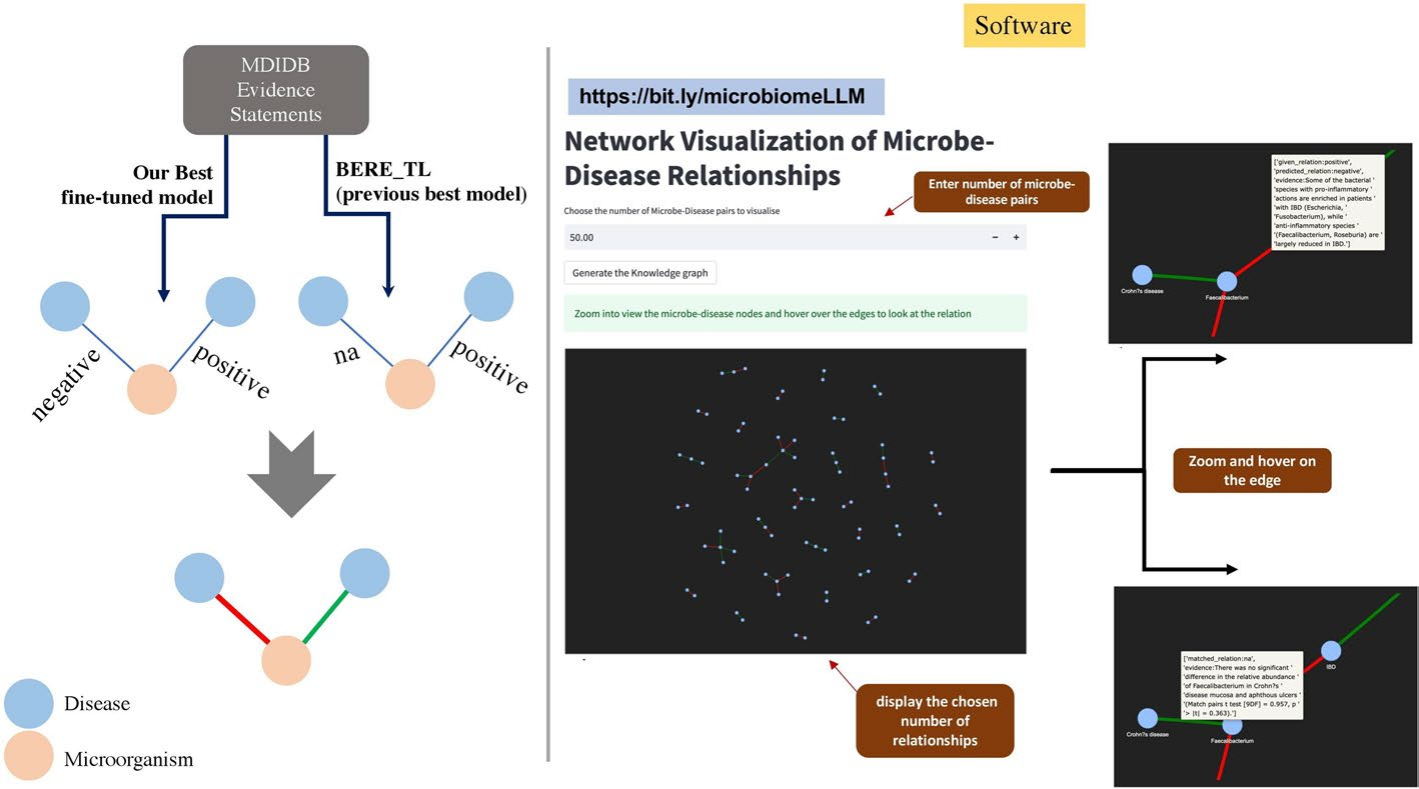
Comparing the MDIDB knowledge base generated using BERE (TL) model versus our prediction model

### Comparison of outputs of our approach using a web-based solution

In the study [24], the authors utilized their best-performing model ($BERE_{TL}$) on a large corpus of text to extract disease-microbiome relationships, which they subsequently released as the *MDIDB* database.

We aimed to compare the outputs generated by our model with those in the *MDIDB* database. To accomplish this, we devised a straightforward graph and visualization strategy, as illustrated in the first panel of Fig. 7. The process involved running both models on the original set of evidence statements used in MDIDB and comparing the resulting graphs. In our graph representation, nodes correspond to diseases or microbes, while edges represent established relationships between them. Nodes are colored green when both algorithms agree on the nature of the relationship, and red when they disagree. We also developed a web application for this which is accessible on our supplementary website [see Additional file 1]. The user interface of the tool allows users to select the number of edges they wish to visualize from the larger graph. After specifying, for example, 50 edges in the provided text box, users can click the “generate knowledge graph” button to display the corresponding knowledge graph. Zooming and hovering over the edges of the graph provide information on the differences in predictions between the two models, including evidence text, for both red and green nodes (as depicted in Fig. 7). This approach aims to provide expert researchers with a more comprehensive understanding of the performance of the different models.

## Discussions

In this paper, we address several crucial aspects concerning the utility of pre-trained language models and their applicability to relevant challenges in the biomedical domain. Specifically, we focus on the task of extracting disease-microbe relationships from scientific publications. To approach this problem, we frame it as a relation extraction task, enabling us to explore the potential of various language models in generative and discriminative paradigms. Our initial investigation involves assessing the capability of generative models, namely GPT-3, BioGPT, and BioMedLM, in a zero-shot or few-shot setting. We sought to determine if these models can perform well on the task with minimal fine-tuning or data preparation. However, we find that their results are of poor quality, highlighting the need for domain-specific adaptations to enhance their usefulness.

Interestingly, we discover that GPT-3 performs the best when fine-tuned. Subsequently, we explore the performance of discriminative models, specifically the BERT-based models and their domain-specific adaptations such as BioMegatron, PubMedBERT, BioClinicalBERT, and BioLinkBERT. As expected, fine-tuning these models yields state-of-the-art results for the task. We also observe that the quality of the training data significantly influences the accuracy improvements achieved. Our work serves as a foundation for further research on adapting and leveraging language models in the field of biomedicine. In conclusion, we have demonstrated that language models in both generative and discriminative settings are viable candidates for fine-tuning and constructing models that yield SOTA results for the microbiome-disease relationship extraction task.

There are several avenues for future exploration. For instance, investigating a broader range of models, including Galactica [40], LLaMA [41], GPT-4 [42], etc., could provide valuable insights for these tasks. Additionally, as we are in the era of models like ChatGPT [43], it would be interesting to explore the possibility of fine-tuning similar conversational models. As a preliminary experiment, we briefly examined ChatGPT using their publicly available service, and the results can be found in the supplementary website [see Additional file 1] of this paper. While the initial findings appear promising, we observed that the model produced different outputs for the same prompt, raising concerns about the reliability of the generated responses. These observations align with our experiences during the fine-tuning of GPT-3, indicating the need for further refinement in this area. Such investigations could lead to exciting new research directions.

Furthermore, we identify limitations in entity recognition and normalization within the GSC dataset. Addressing these issues requires additional work to refine the end-to-end pipeline and build accurate and trustworthy knowledge bases. Another important aspect of this research is that once reliable knowledge bases are established, they can serve as a foundation for formulating hypotheses regarding potentially new disease-microbe associations, thus fostering new knowledge and discoveries [44, 45]. Previous studies, such as those conducted by [46, 47], have explored similar approaches. In addition, while we considered the current work purely as a NLP task, augmenting with other heterogeneous knowledge networks (such as in [48, 49]) can further improve the prediction ability of the models.

## Supplementary Information

**Additional file 1:** Supplementary material.

## Data Availability

All data, model and code used in this project is available at the supplementary companion website https://bit.ly/microbiomeLLM

## Abbreviations

GPT: Generative Pre-trained Transformer
BERT: Bidirectional Encoder Representations from Transformers
MADET: Microbiomics of Anticancer Drug Efficacy and Toxicity
HMDAD: Human Microbe-Disease Association Database
NLP: Natural Language Processing
NER: Named Entity Recognizer
LLMs: Large Language Models
LSTM: Long Short-Term Memory
GSC: Gold Standard Corpus
SSC: Silver Standard Corpus
CC: Commercial-Collection
GPU: Graphics Processing Unit
DDI: Drug-Drug Interaction
MDIDB: Microbe-Disease Interactions Database
TL: Transfer Learning
LLaMA: Large Language Model Meta AI
SOTA: State-Of-The-Art
