# Supplementary material for: Leveraging pre-trained language models for mining microbiome-disease relationships
Source: BMC Bioinformatics. 2023 Jul 19;24:290. doi: 10.1186/s12859-023-05411-z (PMC10357883; doi:10.1186/s12859-023-05411-z)
Supplement: Supplementary file 1 — Additional file 1: Supplementary material. [file 12859_2023_5411_MOESM1_ESM.pdf]

RESEARCH

Supplementary: Leveraging pre-trained language models for mining microbiome-disease relationships

Nikitha Karkera[4], Sathwik Acharya[1,3] and Sucheendra K. Palaniappan[1,2,4*]

*Correspondence: sucheendra@sbi.jp
[1]The Systems Biology Institute, Tokyo, Japan
[2]Iom Bioworks Pvt Ltd., Bengaluru, India
Full list of author information is available at the end of the article

1 Supplementary

1.1 Supplementary Website

All data, model and code used in this project is available at the supplementary companion website https://bit.ly/microbiomeLLM

1.2 Prompts for Zero shot learning

PROMPT

Given the meaning of the labels, answer the following question with the appropriate label.

positive: This type is used to annotate microbe–disease entity pairs with a positive correlation, such as microbe will cause or aggravate the disease, the microbe will increase when disease occurs.
negative: This type is used to annotate microbe disease entity pairs that have a negative correlation, such as microbe can be a treatment for a disease, or microbe will decrease when disease occurs.
relate: This type is used when a microbe disease entity pair appears in the instance and described they are related to each other without additional information
NA: This type is used when a microbe disease entity pair appears in the instance, but the relationship of these two entities has not been described as positive, negative, or relate

based on the above description, evidence is as follows:

The symbiotic therapy involving Bifidobacterium longum and Synergy 1 has been shown to have benefits to both Crohn's disease (CD) and Ulcerative colitis (UC) patients.

Q: What is the relationship between Bifidobacterium longum and Ulcerative colitis (UC)?

A:

1.3 Prompt for few shot learning

PROMPT
Given the meaning of the labels, answer the following question with the appropriate label.

positive: This type is used to annotate microbe–disease entity pairs with a positive correlation, such as microbe will cause or aggravate the disease, the microbe will increase when disease occurs.
negative: This type is used to annotate microbe disease entity pairs that have a negative correlation, such as microbe can be a treatment for a disease, or microbe will decrease when disease occurs.
relate: This type is used when a microbe disease entity pair appears in the instance and described they are related to each other without additional information
NA: This type is used when a microbe disease entity pair appears in the instance, but the relationship of these two entities has not been described as positive, negative, or relate

based on the above description, evidence is as follows:

<Evidence with microbe and disease relation>

Q: What is the relationship between <microbe> and <disease>?
A: positive or negative or relate or na

Two examples in above format for each class

+

<Evidence with microbe and disease relation>
Q: What is the relationship between <microbe> and <disease>?
A:

Inference Prompt

1.4 Data-set for fine tuning: Considerations for improved accuracy

1.4.1 Some examples of mislabeled data in the original GSC data set

- (LAD - Pseudomonas aeruginosa) ”A unique species detected in LAD was Pseudomonas aeruginosa, a bacterium not typically harbored in subgingival plaque, that is associated with severe infections in immunocompromised hosts including LAD patients.” - Finally annotated as ”relate”
- (ASD- Clostridium) ”Because this antibiotic is not absorbed in the gastrointestinal tract and its spectrum of action covers Gram-positive bacteria such as Clostridium, it is possible that these microorganisms play a role in ASD development, especially as vancomycin discontinuation led to a reversion of ASD symptoms.” - Finally annotated as ”positive”
- (Ulcerative Colitis - Clostridiales Ruminococcaceae) ”For example, Clostridiales Ruminococcaceae, the second most important taxon in the RFC model, significantly more abundant in Ent+ individuals, has been found to be un-

derrepresented in individuals suffering from Crohn?s Disease and Ulcerative Colitis." - Finally annotated as "negative"

1.5 Model metrics considered for performance measures

We use the following metrics to measure the performance of the large language models. These metrices were in line with previous work by Wu u.a. (2021).

1.5.1 Accuracy:

Accuracy is a commonly used metric to evaluate the overall performance of a machine learning model. It measures the proportion of correct predictions made by the model over the total number of predictions. It is calculated using the formula:

$$\text{Accuracy} = \frac{\text{True Positives} + \text{True Negatives}}{\text{Total Number of Samples}}$$

1.5.2 Precision:

Precision is a metric that measures the proportion of true positive predictions made by the model over the total number of positive predictions. In other words, it quantifies the accuracy of positive predictions, indicating how often the model correctly identifies positive instances. It is calculated using the formula:

$$\text{Precision} = \frac{\text{True Positives}}{\text{True Positives} + \text{False Positives}}$$

1.5.3 Recall:

Recall, also known as sensitivity or true positive rate, measures the proportion of true positive predictions made by the model over the total number of actual positive instances. It quantifies the model's ability to correctly identify positive instances and is particularly relevant when the cost of false negatives (missed positives) is high. Recall is calculated using the formula:

$$\text{Recall} = \frac{\text{True Positives}}{\text{True Positives} + \text{False Negatives}}$$

1.5.4 F1 Score:

The F1 score is a metric that combines precision and recall into a single value. It provides a balanced measure of a model's performance, taking into account both false positives and false negatives. It is calculated using the formula:

$$\text{F1 Score} = \frac{2 * \text{Precision} * \text{Recall}}{\text{Precision} + \text{Recall}}$$

All of the mentioned metrics are calculated using a weighted approach to address the class imbalance observed in the dataset. The weighted precision is determined by averaging the precision of each class, with weights assigned based on the number of samples in each class. Likewise, the weighted recall and weighted F1-score are computed following the same principle.

Author details
[1]The Systems Biology Institute, Tokyo, Japan. [2]Iom Bioworks Pvt Ltd., Bengaluru, India. [3]PES University, Bengaluru, India. [4]SBX Corporation, Tokyo, Japan.

References
